# Potential of green tea extract to suppress colorectal polyp development in patients with familial adenomatous polyposis: a double-blind, randomized controlled trial Japan Familial Adenomatous Polyposis Prevention Study (J-FAPP Study I)

**DOI:** 10.1186/s41021-026-00354-2

**Published:** 2026-03-02

**Authors:** Hideki Ishikawa, Michihiro Mutoh, Tetsuro Yamane, Keiji Wakabayashi, Keiji Hirata, Takeo Iwama, Tomiyo Nakamura, Naohiro Tomita, Yutaka Matsuyama, J-FAPP Study I Group

**Affiliations:** 1 Department of Molecular-Targeting Prevention, Graduate School of Medical Science, Kyoto Prefectural University of Medicine, Kyoto, Japan; 2 Department of Molecular-Targeting Prevention, Graduate School of Medical Science, Kyoto Prefectural University of Medicine, Kawaramachi-Hirokoji, Kamigyo-ku, Kyoto, 602-8566 Japan; 3 Matsushita Memorial Hospital, Kyoto, Japan; 4 Graduate Division of Nutritional and Environmental Sciences, University of Shizuoka, Shizuoka, Japan; 5 Department of Surgery 1, University of Occupational and Environmental Health, Kitakyushu, Japan; 6 The Department of Digestive Tract and General Surgery, Saitama Medical Center, Saitama Medical University, Saitama, Japan; 7 Research Institute for Food and Agriculture, Ryukoku University, Kyoto, Japan; 8 Division of Cancer Treatment, Toyonaka Municipal Hospital, Osaka, Japan; 9 Department of Biostatistics, School of Public Health, Graduate School of Medicine, The University of Tokyo, Tokyo, Japan

**Keywords:** Chemoprevention, Green tea extract, Randomized controlled trial, Familial adenomatous polyposis

## Abstract

**Background:**

Familial adenomatous polyposis (FAP) is an autosomal dominantly inherited disease that results in the development of more than 100 polyps, premalignant lesions, in the colorectum. Therefore, patients with FAP are a high-risk group for colorectal cancer (CRC). The only standard method of preventing CRC is total colectomy. Thus, alternative methods to prevent the development of CRC are desired by patients. Epidemiological and animal studies suggested that green tea and its extracts, such as (−)-epigallocatechin gallate, may have the potential to prevent cancer development.

**Results:**

In the present study, we evaluated the suppressive effects of green tea extract (GTE) on suppress colorectal polyps in FAP and conducted a double-blind clinical trial. Eighty patients were randomly assigned to the GTE group (1.5 g/day for 2 years) and an equal number were assigned to the placebo group. The primary endpoint of colon polyp enlargement tended to be reduced in the GTE group compared with the placebo group, with a risk ratio of 0.43 (95% confidence interval 0.12–1.49; *p* = 0.16).

**Conclusions:**

Given that the risk ratio was less than 0.5 and few adverse events were observed, we believe that further research using GTE after calculating the necessary sample size is calculated on the based of this study, should be considered in a future large-scale clinical trial.

## Introduction

Familial adenomatous polyposis (FAP) is an autosomal dominantly inherited disease characterized by the development of 100 or more neoplastic lesions (adenomas and carcinomas) in the colorectum, leading to early-onset colorectal cancer (CRC) [1]. Previously, the only method for preventing CRC was total colectomy around the age of 20. Recently, a new treatment, intensive downstaging polypectomy (IDP), has been developed to avoid surgery by aggressively removing colonic polyps endoscopically, which resect all polyps as much as possible, usually hundreds of polyps, in around 90 minutes [2]. IDP is now being performed at many facilities in Japan. However, IDP requires advanced endoscopic techniques, places a heavy burden on endoscopists due to the need to remove numerous polyps, and there remaining risk of not to removing rapidly growing polyps. Therefore, even after the development of IDP treatment, it is important to develop methods to inhibit colonic polyp progression and reduce their size.

Chemoprevention research into FAP using medications to prevent the progression of colorectal polyps has included the administration of aspirin, nonsteroidal anti-inflammatory drugs (NSAIDs), vitamin C, green tea extract (GTE), and dietary fiber. The NSAID sulindac has shown promise in reducing colorectal adenomas in clinical trials [3], but serious side effects with long-term administration have prevented its practical use. Selective cyclooxygenase-2 (COX-2) inhibitors, which are thought to cause less gastrointestinal mucosal damage and bleeding, have been deemed unsuitable for chemoprevention due to their cardiovascular toxicity with long-term administration [4].

Meanwhile, green tea drinkers have been reported to have a lower incidence of gastrointestinal cancers. Observational epidemiological studies have suggested that green tea may have the potential to prevent cancer [5–7]. It contains large amounts of polyphenols (mainly epigallocatechin gallate [EGCG]), which possesses antioxidant, antimutagenic, and antipromoting effects, making them promising candidate chemopreventive agents. It has also been shown to be effective in preventing the development of rodent CRC induced by chemical carcinogenesis [8–10] and to suppress the development of intestinal tumors in Min mice, which have an abnormality in the *Apc* gene, an animal model for FAP [11]. In a single-arm clinical trial, GTE was administered to five patients with FAP, and there was a significant reduction in adenomas in the remaining colorectal mucosa after 4 years of administration (Personal communication from Dr. Yamane).

Based on these results, we conducted a double-blind clinical trial using GTE to suppress colorectal polyps in patients with FAP. Recently, there has been little research into the use of GTE in preventing CRC, but it is now a good time to design new clinical trials.

## Materials and methods

### Study design

This study was a double-blind, randomized, multicenter trial using GTE at 26 medical institutions in Japan. The study enrollment period was from August 28, 2000, to November 22, 2003. Clinical trial registration was not conducted because administration of the test food had ceased around 2006, when mandatory trial registration began.

Patients were randomly assigned 1:1 to two groups, a GTE group and a placebo (NF) group, using a block randomization method. Allocation factors were the facility and whether or not they had undergone colorectal surgery.

### Participants

Participants were men and women aged 16 years or older with 100 or more adenomas in the colorectum or a pathogenic variant in the *APC* gene identified by germline genetic testing. Patients were those who had not undergone colorectal surgery or had residual colon after colectomy.

Exclusion criteria included those with a history of hematemesis or ascites, difficulty with oral intake, a history of green tea allergy, severe anemia (Hb 10 mg/dl or less), a malignant disease, breastfeeding, pregnant women, and those willing to become pregnant. This trial was supported by two FAP patient groups in Japan and the Japanese Society for Hereditary Tumors (JSHT). These groups made the ongoing trial known to patients and gastroenterologists. If a patient wished to participate in the trial, consent was obtained from the patient’s attending physician at the medical facility where the patient was treated, and approval was obtained from the facility’s ethical review committee. After this, researcher Ishikawa and a clinical research coordinator visited the patient’s medical facility, conducted an interview, fully explained the details of the trial, and obtained informed consent and written assent.

The target number of cases was 100. As there has been no research to date on the effect of GTE on polyp reduction in patients with FAP, which served as the basis for calculating the required number of cases, and it was difficult to calculate the required number of cases, so 100 cases was set as the target number of cases considering feasibility.

### Test food

**Fig. 1:**
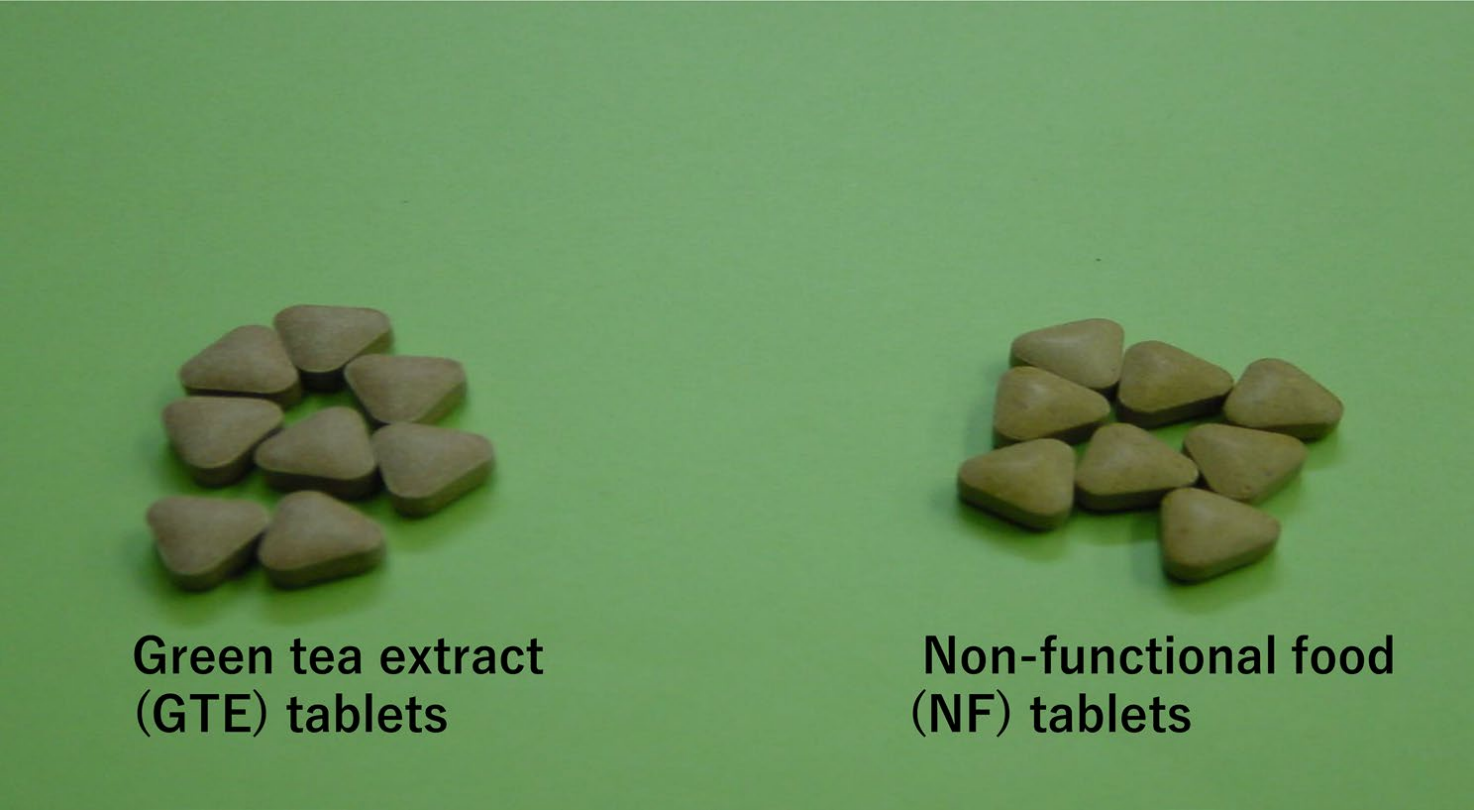
Appearance of test food tablets. The tablets on the left show the appearance of green tea extract (GTE) and those on the right show the appearance of non-functional food (NF). Both were similar in shape and color

The test food used in this study was provided by Taiyo Kagaku Co., Ltd. GTE was administered as six tablets per day after each meal, equivalent to 959.4 mg of GTE (Fig. 1). The catechin content was 501 mg per 6 tablets. High-performance liquid chromatography (HPLC) analysis revealed the following catechin concentrations in one tablet: 3.1 mg of gallic acid, 6.7 mg of (+)-gallocatechin, 10.5 mg of (−)-epigallocatechin, 2.1 mg of (+)-epigallocatechin gallate, 40.7 mg of (−)-epicatechin, 10.0 mg of (−)-gallocatechin gallate, 3.0 mg of (−)-epicatechin gallate, 7.4 mg of (−)-catechin gallate, 0.0 mg. The total concentration of the nine catechins was 83.5 mg per tablet. This amount was equivalent to approximately 10 cups of Japanese tea. The tablets were decaffeinated, with only 0.1 mg per tablet. The planned dosage was manufactured in a single lot on August 18, 1999.

The placebo tablets (NF tablets) were lactose-based and indistinguishable in appearance from the GTE tablets. Each tablet contained 250 mg and was administered six times daily after each meal. These tablets were placed in a bottle and given to patients, who were to take the test food for 2 years.

### Survey items

Prior to the start of the intervention, participants’ height, weight, oral medications, family history, blood tests (blood count, biochemistry), and previous FAP treatment history were recorded. A 3-day continuous dietary record survey [12] was conducted to determine dietary habits, and lactate threshold measurements using a step test were performed to assess physical fitness.

Smoking status was determined for participants who were currently smoking at the time of study entry. Drinkers were defined as those who drank alcohol between once a week and daily.

The primary endpoint was the change in colonic polyps from the lower sigmoid colon to the rectum between pre- and post-treatment for 2 years using colonoscopy. Images were individually assessed by three endoscopists (Toru Otani, Nobuhisa Gondo, and Tetsuro Yamane) who had not performed the endoscopies of the study participants, blinded to the allocation results, as either increased, unchanged, or decreased. In the event of conflicting assessments, the results from the larger number of participants were used.

Photographing was performed as follows: as a standard examination, an endoscope was inserted into the cecum or small intestine to observe the entire colon. Indigo carmine dye was thoroughly sprayed from 20 cm from the anal verge to the anal side. The patient was positioned on the left lateral side, with the fluid level below the screen. Air was introduced to sufficiently expand the intestinal tract until it became cylindrical and wrinkle-free. The endoscope was positioned in the center of the lumen, and photographs were taken while being removed approximately 1 cm at a time. In principle, no endoscopic treatments were performed within 20 cm of the anus during the 2-year study period. Any polyps requiring removal were to be removed after the second-year photographic recording. However, if any polyps requiring removal were identified during the 2-year intervention period, they were to be removed and their removal recorded.

The secondary endpoints included adverse events, the occurrence of colorectal cancer outside the evaluation site, and the occurrence of tumors in other organs.

### Statistical processing

All study data were entered into an Excel file, fixed, and then keyed open by a clinical statistician (Yutaka Matsuyama). The analysis was performed using IBM statistical software SPSS (ver. 20). The primary analysis was performed on all registered cases who were evaluated by colonoscopy in the second year of the population. Relative risk was calculated as a risk ratio and the 95% confidence interval was calculated. When 0 was included, a chi-square test was calculated using Fisher’s exact probability. A *p*-value of 5% or less was considered significant.

## Results

**Fig. 2:**
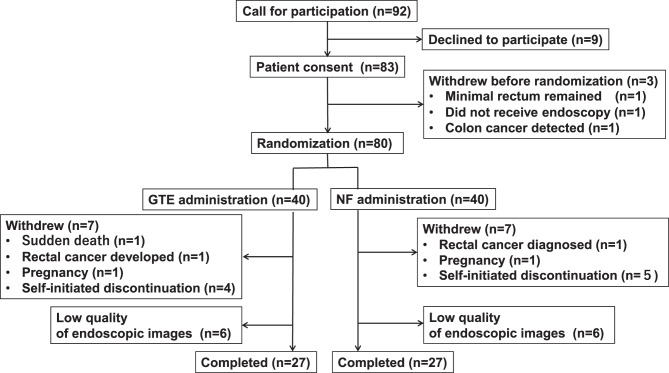
A flow chart of the participants. After providing informed consent, the participants were randomly divided into green tea extract (GTE) group a (left side) and non-functional food (NF) group B. The number of participants and the numbers of patients who withdrew are shown in the solid frames

Figure 2 shows a participant flow chart. Ninety-two patients with FAP were invited to participate, but nine declined. Of the 83 (90%) patients with FAP who consented, one patient did not undergo colonoscopy after providing consent, one patient was found to have minimal rectal tissue remaining, and one patient was diagnosed with CRC during colonoscopy. The remaining 80 patients were randomly assigned to one of two groups after colonoscopy.

Forty patients were assigned to the GTE group, and 40 to the placebo NF group. Seven patients discontinued treatment. In the GTE group, the reasons for discontinuation included rectal cancer diagnosed after rectal bleeding in the first month, sudden death during sleep in the 23rd month, pregnancy, and self-initiated discontinuation in the 4 cases. In the placebo NF group, the reasons for discontinuation included rectal cancer development in the 9th month, pregnancy, and self-initiated discontinuation in the 5 cases. Photographs were not retrieved from 5 patients in the GTE group and 1 patient in the placebo NF group, and photographs of 1 patient in the GTE group and 5 in the placebo NF group were of insufficient quality. Twenty-seven patients in the GTE group and 27 in the placebo NF group underwent evaluation at the final photograph evaluation meeting.

Table 1 shows the background characteristics of the 54 patients who were assessed in the final photograph evaluation meeting. The placebo NF group tended to have slightly more males, be slightly younger, and have a higher prevalence of previous colorectal surgery.

**Table 1:** Participant background

	NF group (*n* = 27)	GTE group (*n* = 27)
Age (year)*	38.4 ± 15.5	44.9 ± 13.2
Sex (male, n)	18 (66.7%)	12 (44.4%)
BMI (kg/m^2^)*	21.9 ± 6.9	20.4 ± 9.9
Weight (kg)*	59.7 ± 9.9	56.7 ± 9.8
Smoker (n)^#^	9 (33.3%)	5 (18.5%)
Drinker (n)^$^	6 (22.2%)	13 (48.1%)
*APC*: Pathogenic variant-positive individuals (n)	20 (74.1%)	20 (74.1%)

*Mean ± standard deviation

^#^Smoker: currently smoking at the time of study entry

^$^Drinker: drank alcohol between once a week and daily

Table 2 shows a comparison between the GTE and placebo NF groups. The primary endpoint of colorectal polyp enlargement tended to be reduced in the GTE group compared with the placebo NF group with a risk ratio of 0.43 (95% confidence interval 0.12–1.49; *p* = 0.16). Subgroup analysis showed a tendency for fewer patients with colorectal polyp enlargement in the GTE group, although this difference was not significant, in men, patients aged 35 years or older, or patients with a history of colorectal surgery.

**Table 2:** Effect of GTE on colonic polyp growth in the second year

	NF group	GTE group	Risk ratio (95% confidence interval)	p-value
Total (increase/total)	7 (25.9%)/27	3 (11.1%)/27	0.43 (0.12–1.49)	p = 0.16
Sex				
Male	5 (27.8%)/18	0 (0.0%)/12		p = 0.07
Female	2 (22.2%)/9	3 (20.0%)/15	0.90 (0.18–4.40)	p = 1
Age				
34 or less	3 (25.0%)/12	2 (28.6%)/7	1.14 (0.25–5.26)	p = 1
35 or over	4 (26.7%)/15	1 (5.3%)/19	0.19 (0.02–1.51)	p = 0.15
Smoking status				
Never & ever smoker (n)	5 (27.8%)/18	3 (13.6%)/22	0.49 (0.14–1.78)	p = 0.43
Smoker (n)	2 (22.2%)/9	0 (0.0%)/5		p = 0.51
Drinking status				
Never & ever drinker (n)	5 (23.8%)/21	1 (7.1%)/14	0.30 (0.04–2.30)	p = 0.37
Drinker (n)	2 (33.3%)/6	2 (15.4%)/13	0.46 (0.08–2.54)	p = 0.56
History of colorectal surgery				
−	6 (40.0%)/15	1 (11.1%)/9	0.28 (0.04–1.95)	p = 0.19
+	1 (8.3%)/12	2 (11.1%)/18	1.33 (0.14–13.1)	p = 1
*APC*: Pathogenic variant				
−	2 (28.6%)/7	0 (0.0%)/7		p = 0.46
+	5 (25.0%)/20	3 (15.0%)/20	0.60 (0.17–2.18)	p = 0.69

Although the causal relationship with this study is unclear, one patient in the GTE group died suddenly. One patient in each group developed CRC. Other than these, no serious adverse events requiring hospitalization were observed in either group.

## Discussion

This study was the first double-blind clinical trial using GTE in patients with FAP to evaluate colorectal polyp suppression as an endpoint. The GTE group showed a strong reduction in the risk of colon polyp growth, with a risk ratio of less than 0.5 (*p* = 0.16), but the small sample size made the results insignificant.

Yamane et al., our collaborators, previously conducted a single-arm intervention clinical trial using GTE. Although this study was an intervention trial with a small number of patients (6 subjects) and no control group, it suggested a tendency toward a reduction in rectal adenoma growth (personal communication from Dr. Yamane). Our study also showed a similar result with a tendency for GTE to inhibit colorectal polyp growth. The lack of significant results in this study may be due to a lack of evaluable patients due to the limited number of participants, with allocation at 80 for evaluation.

Given that the risk ratio was less than 0.5 and that few adverse events were observed, we believe that further research using GTE should be considered, calculating the necessary sample size on the basis of the results of this study and ensuring the necessary number of subjects for statistical purposes.

Many clinical trials of cancer prevention in patients with FAP have used nonsteroidal anti-inflammatory drugs (NSAIDs), particularly sulindac, as chemopreventive agents. However, these studies were not able to show a risk ratio of 0.5 or less. Our previous double-blind, randomized clinical trial using aspirin [13] reduced the incidence of colorectal polyps of 5 mm or larger, with an adjusted odds ratio of 0.37（95% confidence interval:0.21–0.68）. It is assumed that GTE may have a similar effect. Furthermore, important points for a chemopreventive agent in clinical trial are i) the absence of serious side effects; ii) a substance that is relatively inexpensive; and iii) a simple and non-invasive administration method. As in the case of selecting chemopreventive candidates, a large amount of evidence of efficacy in epidemiological studies is needed. GTE meets all of these points, inferring that further studies using GTE are worthwhile.

In addition, it is important for clinical trials to have a clear mode of action through basic research and to demonstrate proof of concept in clinical trials. The mechanism by which GTE inhibits colon tumors is thought to be due to the effects of catechins, such as epigallocatechin gallate, the main component of GTE. Catechins are known for their strong antioxidant, radical-scavenging, and enzyme-modifying properties, and numerous cell and animal studies have reported their effectiveness. For example, administration of EGCG or GTE has been reported to have a significant inhibitory effect on duodenal carcinogenesis in N-ethyl-N’-nitro-N-nitrosoguanidine-treated mice [8], colorectal carcinogenesis in azoxymethane-treated rats [9], and gastric carcinogenesis in N-methyl-N-nitrosourea-treated rats [10]. The mechanisms behind these effects have been suggested to involve the suppression of 8-OHdG production, an indicator of DNA damage [14].

This study had three limitations: i) the small number of cases evaluated, due to the limited number of participants, with an allocation of 80. In other words, the exclusion of approximately one-third (26) of the 80 randomized participants from the analysis inherently introduces a risk of bias. However, there was not much difference in the dropout rate between the two groups, and comparability between the two groups was considered to be maintained to a certain extent. In addition, the high number of withdrawals and indeterminable endoscopic images could also be affected; and ii) limitations of the testing equipment. When this study was conducted in 2000 to 2003, endoscopic equipment and pretreatment methods were not yet fully developed, resulting in less than ideal conditions and poor-quality images. iii) The limited level of medical care in Japan at the time may have led to inconsistent patient management. Clinical guidelines were not available at the time, and many patients did not receive appropriate surveillance. This may have contributed to the lack of uniform patient management during the intervention period, potentially contributing to hidden factors. Currntly, intensive endoscopic downstaging polypectomy (IDP) is widely performed and many patients receive appropriate surveillance, improved equipment has improved patient status, and patients’ conditions are better understood. Given that the above limitations have been improved and that accurate calculations of the number of participants are now possible, we believe that now, a colorectal tumor suppression study using GTE is likely to be successful.

## Conclusion

GTE has the potential to suppress colorectal polyps in FAP, and future large-scale clinical trials are expected to explore this potential.

## Data Availability

Individual participant data will not be made available, but the data that support the findings of this study are available on reasonable request to the corresponding author or the first author.

## References

[1] Iwama T, Tamura K, Morita T, et al. A clinical overview of familial adenomatous polyposis derived from the database of the polyposis registry of Japan. Int J Clin Oncol. 2004;9(4):308–16. 15375708 10.1007/s10147-004-0414-4

[2] Ishikawa H, Yamada M, Sato Y, et al. Intensive endoscopic resection for downstaging of polyp burden in patients with familial adenomatous polyposis (J-FAPP study III): a multicenter prospective interventional study. Endoscopy. 2023;55(4):344–52. 36216266 10.1055/a-1945-9120 PMC10060053

[3] Burke C, van Stolk R, Arber N, et al. Exisulind prevents adenoma formation in familial adenomatous polyposis. Gastroenterology. 2000;118:A657.

[4] Mukherjee D, Nissen SE, Topol EJ. Risk of cardiovascular events associated with selective COX-2 inhibitors. JAMA. 2001 Aug;286(8):954–59. 10.1001/jama.286.8.954. 11509060 10.1001/jama.286.8.954

[5] Imai K, Suga K, Nakachi K. Cancer-preventive effects of drinking green tea among a Japanese population. Prev Med. 1997 Nov-Dec;26(6):769–75. 10.1006/pmed.1997.0242. 9388788 10.1006/pmed.1997.0242

[6] Kono S, Ikeda M, Tokudome S, Kuratsune M. A case-control study of gastric cancer and diet in northern Kyushu, Japan. Jpn J Cancer Res. 1988 Oct;79(10):1067–74. 10.1111/j.1349-7006.1988.tb01528.x. 3143695 10.1111/j.1349-7006.1988.tb01528.x PMC5917639

[7] Kato I, Tominaga S, Matsuura A, Yoshii Y, Shirai M, Kobayashi S. A comparative case-control study of colorectal cancer and adenoma. Jpn J Cancer Res. 1990 Nov;81(11):1101–08. 10.1111/j.1349-7006.1990.tb02520.x. 2125036 10.1111/j.1349-7006.1990.tb02520.x PMC5917987

[8] Fujita Y, Yamane T, Tanaka M, Kuwata K, Okuzumi J, Takahashi T, Fujiki H, Okuda T. Inhibitory effect of (−)-epigallocatechin gallate on carcinogenesis with N-ethyl-N’-nitro-N-nitrosoguanidine in mouse duodenum. Jpn J Cancer Res. 1989 Jun;80(6):503–05. 10.1111/j.1349-7006.1989.tb01666.x. 2503469 10.1111/j.1349-7006.1989.tb01666.x PMC5917795

[9] Yamane T, Hagiwara N, Tateishi M, Akachi S, Kim M, Okuzumi J, Kitao Y, Inagake M, Kuwata K, Takahashi T. Inhibition of azoxymethane-induced colon carcinogenesis in rat by green tea polyphenol fraction. Jpn J Cancer Res. 1991 Dec;82(12):1336–39. 10.1111/j.1349-7006.1991.tb01801.x. 1778755 10.1111/j.1349-7006.1991.tb01801.x PMC5918351

[10] Narisawa T, Fukaura Y. A very low dose of green tea polyphenols in drinking water prevents N-methyl-N-nitrosourea-induced colon carcinogenesis in F344 rats. Jpn J Cancer Res. 1993 Oct;84(10):1007–09. 10.1111/j.1349-7006.1993.tb02792.x. 8226273 10.1111/j.1349-7006.1993.tb02792.x PMC5919059

[11] Yamada Y, Mori H. Multistep carcinogenesis of the colon in Apc(Min/+) mouse. Cancer Sci. 2007 Jan;98(1):6–10. 10.1111/j.1349-7006.2006.00348.x. 17052257 10.1111/j.1349-7006.2006.00348.x PMC11159231

[12] Tokudome S, Goto C, Imaeda N, Tokudome Y, Ikeda M, Maki S. Development of a data-based short food frequency questionnaire for assessing nutrient intake by middle-aged Japanese. Asian Pac J Cancer Prev. 2004;5:40–43. 15075003

[13] Ishikawa H, Mutoh M, Sato Y, et al. Chemoprevention with low-dose aspirin, mesalazine, or both in patients with familial adenomatous polyposis without previous colectomy (J-FAPP study IV): a multicentre, double-blind, randomised, two-by-two factorial design trial. Lancet Gastroenterol Hepatol. 2021;6(6):474–81. 33812492 10.1016/S2468-1253(21)00018-2

[14] Lodovici M, Casalini C, De Filippo C, Copeland E, Xu X, Clifford M, Dolara P. Inhibition of 1,2-dimethylhydrazine-induced oxidative DNA damage in rat colon mucosa by black tea complex polyphenols. Food Chem Toxicol. 2000 Dec;38(12):1085–88. 10.1016/s0278-6915(00)00109-5. 11033196 10.1016/s0278-6915(00)00109-5

